# Graphene Oxide and Fluorescent-Aptamer-Based Novel Aptasensors for Detection of Metastatic Colorectal Cancer Cells

**DOI:** 10.3390/polym14153040

**Published:** 2022-07-27

**Authors:** Hang Chen, Shurui Zhang, Yung-Chin Hsiao, Qun Wang, Jau-Song Yu, Wanming Li

**Affiliations:** 1 Experiment Teaching Center of Functional Subjects, College of Basic Medicine, China Medical University, No. 77 Puhe Road, Shenyang North New Area, Shenyang 110122, China; lnfsch@hotmail.com; 2 Department of Cell Biology, Key Laboratory of Cell Biology, Ministry of Public Health, and Key Laboratory of Medical Cell Biology, Ministry of Education, China Medical University, No. 77 Puhe Road, Shenyang North New Area, Shenyang 110122, China; shuruiz@126.com (S.Z.); wqshine16@163.com (Q.W.); 3 Molecular Medicine Research Center, Chang Gung University, Taoyuan 333, Taiwan; hschin@mail.cgu.edu.tw (Y.-C.H.); yusong@mail.cgu.edu.tw (J.-S.Y.); 4 Graduate Institute of Biomedical Sciences, College of Medicine, Chang Gung University, Taoyuan 333, Taiwan; 5 Liver Research Center, Chang Gung Memorial Hospital at Linkou, Taoyuan 333, Taiwan

**Keywords:** aptamer, colorectal cancer cells, graphene oxide, metastatic

## Abstract

Early diagnosis of metastatic colorectal cancer (mCRC) is extremely critical to improve treatment and extend survival. W3 is an aptamer that can specifically bind to mCRC cells with high affinity. Graphene oxide (GO) is a two-dimensional graphitic carbon nanomaterial, which has widely used in constructing biosensors. In this study, we have developed a no-wash fluorescent aptasensor for one-step and sensitive detection of mCRC LoVo cells. It is based on fluorescence resonance energy transfer (FRET) between GO and the W3 aptamer labeled with 5-carboxyfluorescein (FAM). GO can quench the green fluorescence of the FAM-labeled W3 (FAM-W3). In the presence of the target cells, FAM-W3 preferentially binds the target cells and detaches from the surface of GO, leading to the fluorescence of FAM recovery. It was demonstrated that the fluorescence recovery increases linearly in a wide range of 0~10^7^ cells/mL (R^2^ = 0.99). The GO-based FAM-labeled W3 aptasensor (denoted as FAM-W3-GO) not only specifically recognizes mCRC cell lines (LoVo and HCT116), but also sensitively differentiates the target cells from mixed cells, even in the presence of only 5% of the target cells. Furthermore, FAM-W3-GO was applied to detect LoVo cells in human whole blood, which showed good reproducibility with an RSD range of 1.49% to 1.80%. Therefore, FAM-W3-GO may have great potential for early diagnosis of mCRC. This strategy of GO-based fluorescent aptasensor provides a simple, one-step, and highly sensitive approach for the detection of mCRC cells.

## 1. Introduction

Colorectal cancer (CRC) is the third most frequently diagnosed cancer in the world, with approximately 151,030 new cases in the United States in 2022 [1]. Metastasis is the main cause of cancer-related death. Patients with metastatic CRC (mCRC) have poor prognosis with a low five-year survival rate [2]. Therefore, the detection of mCRC cells before cancer progress is significant for the treatment of mCRC patients. At present, the methods used for mCRC cells’ detection are mainly based on specific tumor biomarkers, circulating tumor DNA (ctDNA), or micro RNA in blood [3,4,5]. Those testing have diagnostic value in CRC, but developing highly sensitive and specific methods is needed for the detection of mCRC.

Aptamers are short single-stranded DNA (ssDNA) or RNA oligonucleotides that have the ability to bind target substances with high affinity and specificity. They are generated from randomly synthesized nucleic acid libraries via a process known as systematic evolution of ligands by exponential enrichment (SELEX) [6,7,8]. Aptamers have several characteristics, including high stability, easy synthesis and modification, and low immunogenicity. Compared with antibodies, aptamers are more convenient for conjugation with various biomaterials to yield molecular probes for clinic diagnosis and targeted therapy [9,10,11]. Cell-SELEX, as a modified SELEX, was performed using intact living cells as the target for aptamer selection [12,13]. Aptamers selected via cell-SELEX can bind specifically to their targets on the surface of specific phenotype cells, which can be applied in many ways, including specific cell enriching, cell imaging, and targeted delivery of drugs to specific cells [14,15,16,17].

Graphene oxide (GO) is a two-dimensional graphitic carbon nanomaterial, which has been widely used in the development of biosensors owing to its unique properties including biocompatibility, good water solubility, strong fluorescent quenching ability, and no wash workflow [18,19,20,21]. GO can interact with fluorophore-labeled aptamers by π–π stacking, resulting fluorescence resonance energy transfer (FRET) from the fluorophore to GO in the absence of the target. In the presence of the target, the fluorescence is restored [22]. The change in fluorescence intensity is used to accomplish the detection of targets recognized by aptamers [23,24]. Compared with fluorescent aptamer probes, GO-based fluorescent aptasensors have various advantages, such as a wide linear range, being highly efficient and wash-free, and having good dispersibility in water and low background fluorescence [25,26].

In previous studies, we generated an aptamer W3, which can bind specifically to high mCRC cells. Being conjugated to quantum dots (QDs), W3 can image the target cells of cell lines or tissues from patients with CRC, indicating that it is a perfect probe for the detection of mCRC cells [27]. In this study, we designed a GO-based fluorescent aptasensor (FAM-W3-GO) for one-step and sensitive detection of mCRC cells without washing workflow. In the process of the analysis, GO is linked with FAM-W3 and quenches the fluorescence of FAM. When the target cells are added, green fluorescence will be restored along with the release of FAM-W3. Therefore, a highly sensitive aptasensor for mCRC cell detection is accomplished by the fluorescence change in the absence and presence of target cells. Because of the specific recognition of W3, FAM-W3-GO could be applied in the detection of mCRC cells in complex circumstances. The detection procedure is simple, and one-step targeted detection can be achieved without washing steps. 

## 2. Materials and Methods 

### 2.1. Reagents and Materials

The W3 aptamer (5′-AGCAGCGTGGAGGATAGGGGTCGGAGTGGGTGGTTATGATTGGCTCTTCTGCGCTGC-3′) was synthesized and labeled with FAM at the 5′ end by Sangon Biotechnology Co. Ltd. (Shanghai, China). FAM-W3 was dissolved by Tris-HCl buffer (20 mM, pH 7.4). GO was purchased from the XF NANO, Inc. (Nanjing, China). All solutions used in the research were prepared with deionized water purified from a Milli-Q purification system (Millipore, Bedford, MA, USA).

### 2.2. Apparatus

Fluorescence intensity data were measured on a Hitachi F-4500 fluorescence spectrophotometer (λ excitation/emission = 480/518 nm). All of the fluorescence images of the cells were obtained with an Olympus IX51 fluorescence microscope (Olympus, Tokyo, Japan). The ultraviolet visible (UV/Vis) absorption measurements were recorded on TU-1901 Dual-beam UV/Vis Spectrophotometer (Beijing, China).

### 2.3. Cell Lines and Cell Culture

The human CRC cell line LoVo was used as the target cell. The other cell lines used included the human CRC cell lines, HCT116, HT29, and CL187, and the human embryonic kidney cell line HEK293. LoVo, HCT116, and HEK293 were purchased from the Cell Bank of the Chinese Academy of Sciences (Shanghai, China). HT29 and CL187 were purchased from American Type Culture Collection (ATCC, Manassas, VA, USA). All of the cells were cultured at 37 °C under a 5% CO_2_ atmosphere. The growth medium used for LoVo, HCT116, and HT29 cells was RPMI1640 medium (GIBCO), containing 10% fetal bovine serum (FBS, Invitrogen, Carlsbad, CA, USA) and 100 U/mL penicillin-streptomycin (Sigma-Aldrich, St. Louis, MO, USA). The growth medium for the other cell lines was high-glucose DMEM (GIBCO), containing 10% FBS and 100 U/mL penicillin-streptomycin (Sigma-Aldrich, Darmstadt, Germany).

### 2.4. Preparation of GO-Based Fluorescent Aptasensor

The GO powder was dissolved in deionized water and a solution with a concentration of 0.5 mg/mL was obtained. FAM-W3 was dissolved in 20 mM Tris-HCl buffer and a concentration of 100 μM FAM-W3 was obtained. After that, 1 μL FAM-W3 was added into the 10 μL GO solution, mixed, and then diluted with Tris-HCl buffer to 500 μL FAM-W3-GO solution. The fluorescence emission spectra, including FAM-W3, GO, and FAM-W3-GO solution, were confirmed by UV/Vis absorption spectroscopy.

### 2.5. Fluorescence Labeling Based on FAM-W3-GO

LoVo and HEK293 cells were seeded in six-well plates and cultured overnight. After removal of the medium, the cells were first washed twice with cold phosphate-buffered saline (PBS, pH 7.4) and then incubated with FAM-W3-GO solution at 4 °C in the dark for 30 min, respectively. Next, all cells were fixed with 4% formaldehyde for 15 min, and then stained with 4’, 6-diamidino-2-phenylindole (DAPI) for 5 min to counterstain the nucleus. The cells were examined using fluorescence microscope without washing steps.

### 2.6. Target Cells’ Detection by GO-Based Fluorescent Assay

LoVo cells were cultured for 24 h, collected, and suspended in PBS. Different numbers of LoVo cells (50, 500, 5 × 10^3^, 5 × 10^4^, 5 × 10^5^, and 5 × 10^6^) were incubated with FAM-W3-GO (500 μL final volume) at 4 °C in the dark for 30 min, with a final cell concentration of 0 to 1.0 × 10^7^/mL. After incubation, the LoVo cells were tested with a fluorescence spectrophotometer. The limit of detection (LOD) was calculated according to 3*σ*/*S*, where *σ* and *S* are the standard deviation of the three blank measurements and the slope of the linear equation, respectively.

### 2.7. Specificity Analysis

To examine the specificity of GO-based fluorescent aptasensor, a list of cancer cell lines, including LoVo, HCT116, HT29, CL187, and a normal cell line HEK293, were added into the FAM-W3-GO solution. Each reaction system included 1 × 10^6^ cells and was performed following the same experimental procedures. Besides, HEK293 and LoVo cells were mixed, and then the mixed cell samples were incubated with FAM-W3-GO at 4 °C in the dark for 30 min. After incubation, each sample was detected using flow cytometry (Beckman Coulter, Sykesville, MD, USA). 

### 2.8. Whole Blood Sample Analysis 

Human blood samples were obtained from donations from healthy people. Here, 0.5 mL blood samples were diluted ten times with Tris-HCl buffer (20 mM, pH 7.4). Two groups of LoVo cells (100, 500 cells/100 μL) were spiked in 400 μL of diluted blood samples to reach a final volume of 500 μL, respectively, and then incubated with FAM-W3-GO in the dark for 30 min. After incubation, each sample was tested with a fluorescence spectrophotometer. Diluted blood samples without the target cells were used as a negative control. The relative standard deviation (RSD) and spike recovery were calculated by the calibration plot. 

### 2.9. Statistical Analyses

Each experiment was performed in triplicate. The data were processed using Student’s *t*-test and one-way analysis of variance. The statistical analyses were determined using GraphPad Prism software.

## 3. Results and Discussion

### 3.1. Principle of FAM-W3-GO for Cancer Cell Detection 

**Scheme 1:**
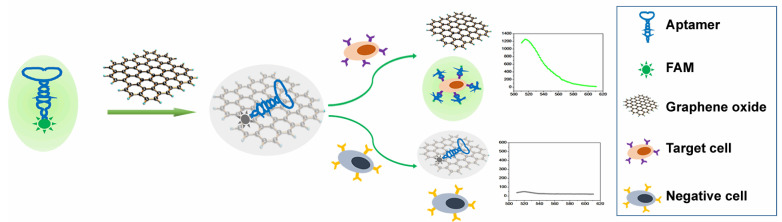
Schematic illustration represents the working principle of the GO-based fluorescent aptasensor assay.

Monoclonal antibodies, aptamers, and small molecule drugs have been applied for the early detection of cancers [28,29,30]. In this study, a fluorescent aptasensor (FAM-W3-GO), consisting of GO and FAM-W3, was designed for the fast, easy, and specific detection of mCRC cells. The principle of FAM-W3-GO for detection of mCRC cells is illustrated in Scheme 1. The W3 aptamer, obtained from cell-SELEX selection using mCRC LoVo cells as the target cells, can specifically bind to mCRC cells with high affinity. In the absence of the target cells, FAM-W3 can be absorbed onto the GO surface via π–π stacking, resulting in efficient fluorescence quenching of FAM. When the target cells are added, FAM-W3 can specifically bind with the target cells and the binding will alter the conformation of FAM-W3, which allows FAM-W3 to stay away from GO, causing the fluorescence recovery. Therefore, the target cells could be detected effectively by monitoring the changes in fluorescence signal of FAM-W3. In contrast, in the presence of negative cells, FAM-W3 cannot bind negative cells and is still absorbed on the surface of GO, thus there is no obvious fluorescence recovery of FAM-W3. Given the strong adsorption and fluorescent quenching ability of GO, FAM-W3-GO has good sensitivity and is easily operated without wash workflow. 

### 3.2. The Feasibility of mCRC Cell Detection Using FAM-W3-GO 

**Figure 1:**
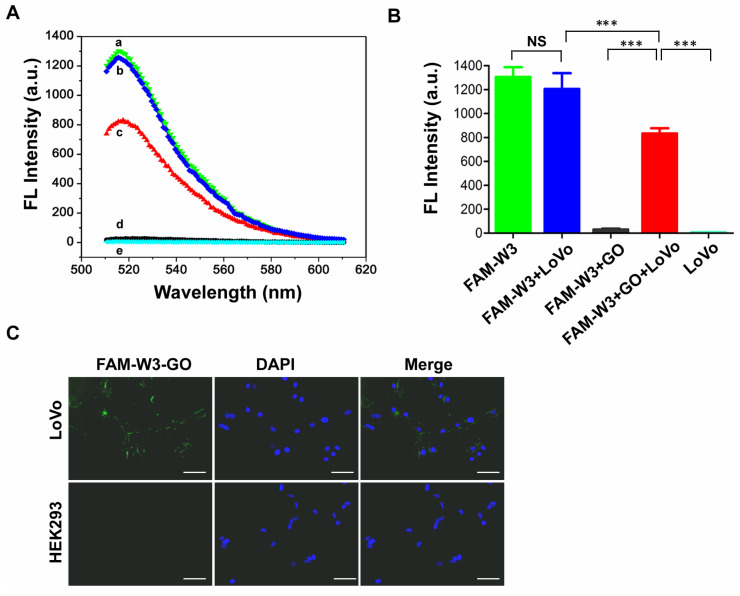
(**A**) The fluorescence spectra of FAM-W3 and LoVo cells at different conditions: (a) FAM-W3, (b) FAM-W3 + LoVo, (c) FAM-W3 + GO + LoVo, (d) FAM-W3 + GO, and (e) LoVo. (**B**) Statistical analysis of fluorescence spectra of FAM-W3 and LoVo cells at different conditions. NS, not significant; ***, *p* < 0.001. (**C**) Fluorescence images of LoVo and HEK293 cells incubated with FAM-W3-GO. The nuclei were stained with DAPI. Scale bar = 50 μm.

To prove the feasibility of mCRC cell detection based on FAM-W3 and GO, the fluorescence intensity at different systems (FAM-W3, FAM-W3 + LoVo, FAM-W3 + GO, FAM-W3 + GO + LoVo, and LoVo cells alone), was measured. As shown in Figure 1A, FAM-W3 exhibited a strong fluorescence intensity at 518 nm (Figure 1A, curve a). In the presence of GO, the fluorescence intensity was remarkably reduced (Figure 1A, curve d), close to the non-fluorescent LoVo cells (Figure 1A, curve e), indicating that GO effectively quenched the fluorescence of FAM when FAM-W3 was adsorbed onto the GO surface. Moreover, when FAM-W3-GO was incubated with targeting LoVo cells, the pre-quenched fluorescence exhibited a significant recovery (Figure 1A, curve c). The results revealed that W3 has stronger binding affinity to targeting LoVo cells than GO. The binding of LoVo cells to W3 allows the W3 aptamer to detach from the surface of GO, and then the fluorescence is restored. However, the fluorescence intensity of FAM-W3 without GO conjugation has no significant change when targeting LoVo cells were added (Figure 1A, curve b). Additionally, statistical analysis of fluorescence intensity is shown in Figure 1B. 

Furthermore, we evaluated the imaging performance of FAM-W3-GO using fluorescence microscope in two cell lines, including mCRC LoVo cells and HEK293 cells. As displayed in Figure 1C, after incubation with FAM-W3-GO, the surface of LoVo cells exhibited obvious green fluorescence without any washing step. Meanwhile, under the same conditions, no fluorescence was found on control HEK293 cells, indicating that the binding between FAM-W3-GO and mCRC LoVo cells was specific and effective. Collectively, FAM-W3-GO could successfully detect the presence of mCRC cells according to the change in fluorescent intensity before and after adding targeting LoVo cells. 

### 3.3. Optical Properties of FAM-W3-GO

**Figure 2:**
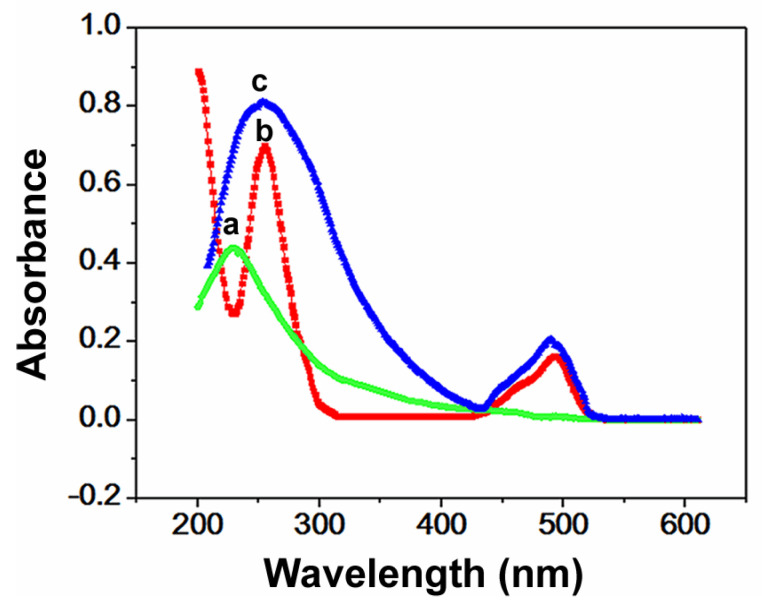
UV-visible absorbance spectra of (a) GO, (b) FAM-W3, and (c) FAM-W3-GO.

To evaluate FAM-W3-GO conjugates, UV/Vis spectra of GO, FAM-W3, and FAM-W3-GO were measured. Figure 2 shows that the characteristic absorption peak of GO appeared at 230 nm (Figure 2, curve a), which is assigned to the π–π transitions of aromatic C-C bonds. UV/Vis spectrum showed the two absorption peaks of FAM-W3 at 260 nm and 503 nm (Figure 2, curve b), which correspond to the DNA sequence and FAM, respectively. After GO was added into the solution of FAM-W3, the absorbance spectrum of GO showed a red shift and the FAM absorption peak was increased at 503 nm compared with that of FAM-W3 alone (Figure 2, curve c), which is due to electron transition between the π–π system and the dyes. The absorption data confirmed the formation of FAM-W3-GO. 

### 3.4. Optimization of Experimental Conditions 

**Figure 3:**
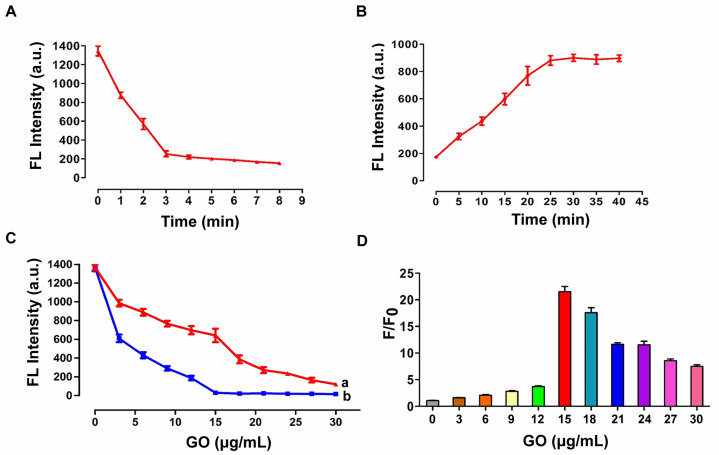
(**A**) Time dependence of the fluorescence quenching degree for 200 nM FAM-W3 caused by GO. (**B**) Time dependence of the fluorescence recovery degree for FAM-W3 in GO solution when LoVo cells were added. (**C**) Effect of GO concentration on the fluorescence intensity of FAM-W3 in the presence (curve a) and in the absence (curve b) of LoVo cells. (**D**) The fluorescence intensity rate (*F/F*_0_) of FAM-W3 is plotted as a function of GO concentration. *F* and *F*_0_ represent the fluorescence intensity of FAM-W3 in the presence and absence of target LoVo cells.

To achieve the best performance of FAM-W3-GO, the fluorescence quenching time, the incubation time for fluorescence recovery, and the GO concentration were investigated. Firstly, GO was added to the solution of 200 nM FAM-W3, and then the fluorescence intensity at 518 nm was measured at different time points (0, 1, 2, 3, 4, 5, 6, 7, and 8 min). As described in Figure 3A, in the presence of GO, FAM-W3 adsorbed to the surface of GO, and the fluorescence intensity was reduced quickly until it tended to a steady-state after 3 min, indicating that it only takes 3 min to reach the quenching equilibrium. This result demonstrated that GO can rapidly quench fluorescence. Furthermore, the incubation time for fluorescence recovery with FAM-W3-GO was optimized. LoVo cells (1 × 10^6^ cells) were added to the solution containing FAM-W3-GO, incubated at 4 °C in the dark for different times (0, 5, 10, 15, 20, 25, 30, 35, and 40 min), and then the fluorescence intensity was measured by fluorescence spectrophotometer. As shown in Figure 3B, with the increasing incubation time, the fluorescence intensity was gradually increased and reached the maximum at 30 min. Therefore, 30 min of incubation time was chosen as the optimum time for the subsequent experiment.

Additionally, the GO concentration was also optimized. Figure 3C shows the relationship between the fluorescence intensity and the concentration of GO with (Figure 3C, curve a) or without (Figure 3C, curve b) LoVo cells, respectively. As shown in Figure 3C, the addition of increasing concentrations of GO (from 0 to 30 μg/mL) led to significant a decrease in the fluorescence intensity. In Figure 3D, it can be seen that the ratio of *F/F*_0_ was gradually increased when the concentration of GO was increased from 0 to 15 μg/mL, and reached the maximum value at the concentration of 15 μg/mL. When GO concentration was greater than 15 μg/mL, the ratio of *F/F*_0_ was decreased. *F* and *F*_0_ refer to the fluorescence intensity of FAM-W3 in the presence and absence of LoVo cells, respectively. Therefore, in the subsequent experiment, the GO concentration was fixed at 15 μg/mL. 

### 3.5. Detection of LoVo Cells with FAM-W3-GO 

**Figure 4:**
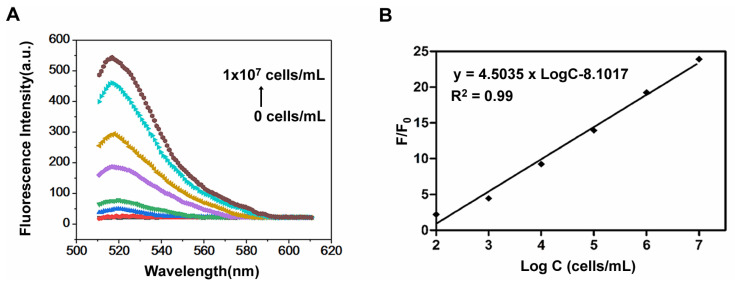
LoVo cells’ detection using a FAM-W3-GO fluorescent aptasensor. (**A**) The fluorescence spectra of FAM-W3-GO after incubation with different concentrations of LoVo cells from 0 to 1 × 10^7^ cells/mL. (**B**) Calibration plot of the fluorescence intensity rate (*F/F*_0_) versus the concentration of LoVo cells.

Under the optimized conditions, fluorescence intensities of different concentrations of LoVo cells were investigated using FAM-W3-GO. Figure 4A shows that the fluorescence intensity increased gradually with the addition of increasing numbers of LoVo cells (0 to 1.0 × 10^7^ cells/mL), indicating that more and more FAM-W3 detached from GO and bound to target cells, resulting in an obvious increase in the fluorescence intensity. Furthermore, a linear relationship in the fluorescence intensity rate (*F/F*_0_) of FAM and the number of LoVo cells in the range of 0~10^7^ cells/mL was obtained (Figure 4B). The regression equation is y(*F/F*_0_) = 4.5035 × LogC-8.1017 with a correlation coefficient of 0.99. Compared with previously reported methods for mCRC cells’ detection, our method can detect LoVo cells in a wider linear range with an LOD of 70 cells/mL (Table 1) [31]. Thus, FAM-W3-GO can be used as an ideal fluorescent aptasensor to detect mCRC cells.

### 3.6. Specificity Analysis of FAM-W3-GO 

**Figure 5:**
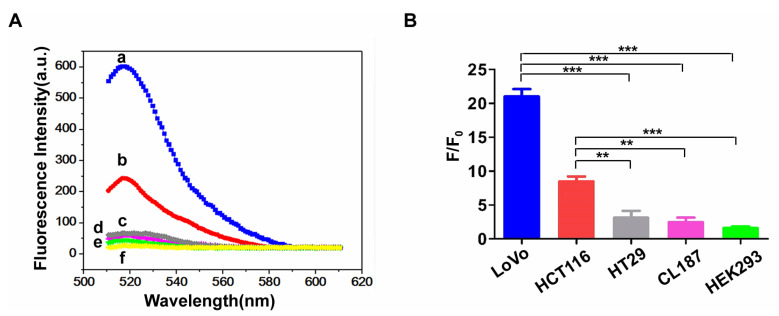
Specificity of FAM-W3-GO fluorescent aptasensor for mCRC cells. (**A**) The fluorescence spectra of different cell lines incubated with FAM-W3-GO: (a) LoVo cells, (b) HCT116 cells, (c) HT29 cells, (d) CL187 cells, (e) HEK293 cells, and (f) PBS. (**B**) The fluorescence intensity rate (*F/F*_0_) of FAM-W3-GO fluorescent aptasensor in the presence of LoVo, HCT116, HT29, CL187, and HEK293 cells, respectively. **, *p* < 0.01; ***, *p* < 0.001.

Several different cells, including LoVo, HCT116, HT29, CL187, and HEK293 cells, were used to test the specificity of FAM-W3-GO for mCRC cells. As expected, targeting LoVo cells incubated with FAM-W3-GO exhibited the highest fluorescence intensity at 518 nm. Besides, HCT116 cells, another mCRC cell line, obtained higher fluorescence intensity than non-metastatic HT29, CL187, and normal HEK293 cells (Figure 5A). The results are in agreement with previous studies finding that the W3 aptamer specifically binds mCRC cells with high affinity [27]. Moreover, Figure 5B showed that ratios of *F*/*F*_0_ in mCRC cells, including LoVo and HCT116 cells, were remarkably higher than those of the control HEK293 cells. The results indicated that FAM-W3-GO exhibits excellent specificity towards mCRC cells. 

**Figure 6:**
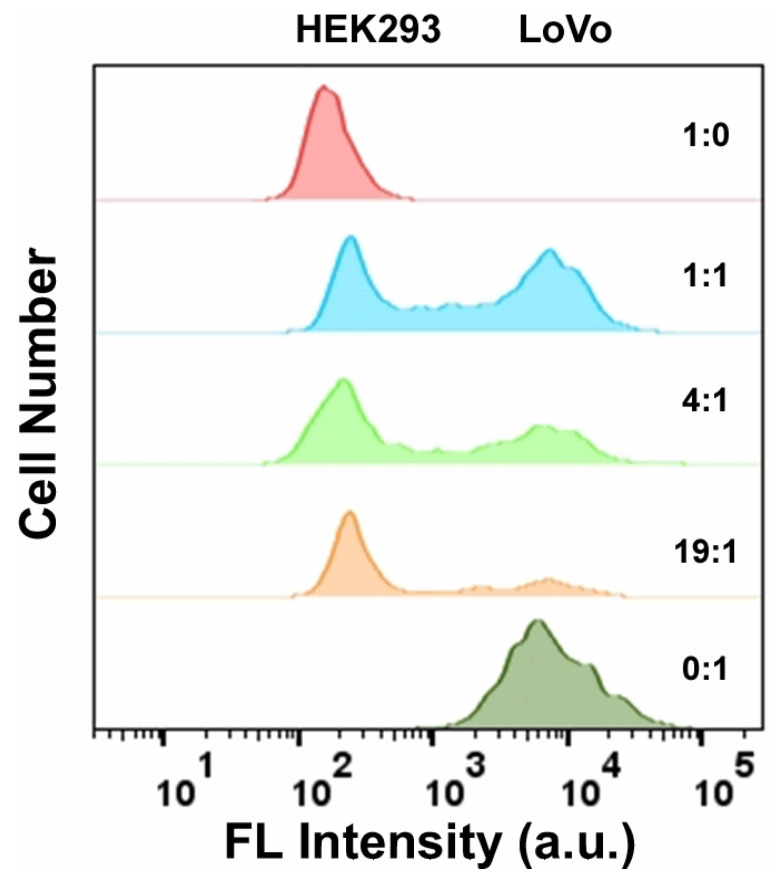
Detection of LoVo cells in mixed cell samples (HEK293/LoVo = 1:0, 1:1, 4:1, 19:1, and 0:1) after incubation with FAM-W3-GO by flow cytometry.

In addition, FAM-W3-GO was used to detect LoVo cells in mixed cell samples. Ratios of LoVo cells to HEK293 cells in mixed cells were 0%, 5%, 20%, 50%, and 100%. Figure 6 shows that decreasing HEK293 cells were observed in the low-fluorescence region, while cell numbers of the high-fluorescence region gradually increased with a rising number of LoVo cells in the cell mixture. It was suggested that FAM-W3-GO enables efficient and specific detection of mCRC cells from mixed cell samples.

### 3.7. Detection of LoVo Cells in Whole Blood Samples Using FAM-W3-GO

To expand the potential application of FAM-W3-GO to detect mCRC cells in real samples, LoVo cells were spiked in the whole blood samples at two final concentrations (100 and 500 cells/500 μL) and incubated with FAM-W3-GO for cell detection. The results are summarized in Table 2. The recoveries in whole blood were from 93.2% to 102.0%, indicating that FAM-W3-GO exhibited good reproducibility and specificity, even in a complex biological sample. The RSDs of recoveries were 1.49% and 1.8%, respectively, suggesting that the assay could be acceptable as a quantitative detection carried out on complex biological samples. 

## 4. Conclusions

We have constructed a GO-based fluorescent aptasensor FAM-W3-GO for washing-free, one-step, and sensitive detection of mCRC cells. This strategy uses GO as the fluorescence nanoquencher and FAM-W3 as the indicator, realizing in vitro detection of target cells. The detection process using conventional fluorescent aptamer probes (W3-QD or FAM-W3) unavoidably undergoes multi-step manipulations, such as repeated sampling and washing. This method, benefiting from GO fluorescence quenching, can reduce the background signal of fluorescence, and the target detection can be achieved by a simple one-step method without repeated washing. Under optimized conditions, the detection range of FAM-W3-GO can be as wide as 0 to 1 × 10^7^ cells with an LOD of 70 cells/mL. In addition, FAM-W3-GO can specifically distinguish between LoVo cells and normal HEK293 cells in mixed cell samples. Moreover, FAM-W3-GO showed good specificity and anti-interference capability, and can successfully detect mCRC LoVo cells in real blood samples, indicating that it holds potential to be a promising probe for mCRC cells’ detection. Therefore, based on its simplicity, no-wash workflow, and excellent sensitivity performance, FAM-W3-GO may be a promising alternative for the detection of mCRC cells. 

## Figures and Tables

**Table 1:** Comparing the linear range and detection limit of different W3 aptamer-based aptasensors.

Detection Method	Linear Range	Detection Limit (/mL)	Reference
Terminal deoxynucleotidyl	23–1.5 × 10^4^	115	[31]
FAM	1.2 × 10^2^–2 × 10^4^	600	[31]
Graphene Oxide	1 × 10^2^–1 × 10^7^	70	This work

**Table 2:** Recoveries and RSDs of LoVo cells at two concentrations (n = 3).

Spiked (Number of Cells/500 μL)	Measured (Number of Cells/500 μL)	Recovery (%)	RSD (%)
100	102 ± 2	102.0 ± 1.5	1.49
500	466 ± 9	93.2 ± 1.7	1.80

## Data Availability

The study did not report any data.

## References

[1] Siegel R.L., Miller K.D., Fuchs H.E., Jemal A. (2022). Cancer statistics. CA Cancer J. Clin..

[2] Van Cutsem E., Köhne C.H., Hitre E., Zaluski J., Chang Chien C.R., Makhson A., D’Haens G., Pintér T., Lim R., Bodoky G. (2009). Cetuximab and chemotherapy as initial treatment for metastatic colorectal cancer. N. Engl. J. Med..

[3] Katelyn N.S., Katherine H.R.T. (2022). Circulating Biomarkers in Breast Cancer. Clin. Breast Cancer.

[4] Barault L., Amatu A., Siravegna G., Ponzetti A., Moran S., Cassingena A., Mussolin B., Falcomata C., Binder M.A., Cristiano A. (2018). Discovery of methylated circulating DNA biomarkers for comprehensive non-invasive monitoring of treatment response in metastatic colorectal cancer. Gut.

[5] Madanat-Harjuoja L.M., Klega K., Lu Y., Shulman D.S., Thorner A.R., Nag A., Tap W.D., Reinke D.K., Diller L., Ballman K.V. (2022). Circulating Tumor DNA Is Associated with Response and Survival in Patients with Advanced Leiomyosarcoma. Clin. Cancer Res..

[6] Tuerk C., Gold L. (1990). Systematic evolution of ligands by exponential enrichment: RNA ligands to bacteriophage T4 DNA polymerase. Science.

[7] Ellington A.D., Szostak J.W. (1990). In vitro selection of RNA molecules that bind specific ligands. Nature.

[8] Liu X., Wang T., Wu Y., Tan Y., Jiang T., Li K., Lou B., Chen L., Liu Y., Liu Z. (2022). Aptamer based probes for living cell intracellular molecules detection. Biosens. Bioelectron..

[9] Jayasena S.D. (1999). Aptamers: An emerging class of molecules that rival antibodies in diagnostics. Clin. Chem..

[10] Yu H., Alkhamis O., Canoura J., Liu Y., Xiao Y. (2021). Advances and Challenges in Small-Molecule DNA Aptamer Isolation, Characterization, and Sensor Development. Angew. Chem. Int. Ed. Engl..

[11] Centane S., Nyokong T. (2022). Aptamer versus antibodies as probe for the impedimetric biosensors for human epidermal growth factor receptor. J. Inorg. Biochem..

[12] Shangguan D.H., Li Y., Tang Z.W., Cao Z.C., Chen H.W., Mallikaratchy P., Sefah K., Yang J.C., Tan W.H. (2006). Aptamer evolved from live cells as effective molecular probes for cancer study. Proc. Natl. Acad. Sci. USA.

[13] Li W.M., Bing T., Wang R., Jin S.H., Shangguan D.H., Chen H. (2021). Cell-SELEX-based selection of ssDNA aptamers for specifically targeting *BRAF V600E*-mutated melanoma. Analyst.

[14] Subjakova V., Oravczova V., Hianik T. (2021). Polymer Nanoparticles and Nanomoyors Modified by DNA/RNA Aptamers and Antibodies in Targeted Therapy of Cancer. Polymers.

[15] Wu L., Wang Y., Xu X., Liu Y., Lin B., Zhang M., Zhang J., Wan S., Yang C., Tan W. (2021). Aptamer-Based Detection of Circulating Targets for Precision Medicine. Chem. Rev..

[16] Li W.M., Zhou L.L., Zheng M., Fang J. (2018). Selection of Metastatic Breast Cancer Cell-Specific Aptamers for the Capture of CTCs with a Metastatic Phenotype by Cell-SELEX. Mol. Ther. Nucleic Acids.

[17] Li W.M., Chen H., Yu M., Fang J. (2014). Targeted delivery of doxorubicin using a colorectal cancer-specific ssDNA aptamer. Anat. Rec..

[18] Biru E.I., Necolau M.I., Zainea A., Iovu H. (2022). Graphene Oxide-Protein-Based Scaffolds for Tissue Engineering: Recent Advance and Applications. Polymers.

[19] Wu J., Jia L., Zhang Y., Qu Y., Jia B., Moss D.J. (2021). Graphene Oxide for Integrated Photonics and Flat Optics. Adv. Mater..

[20] Mao M., Zhang W., Huang Z., Huang J., Wang J., Li W., Gu S. (2021). Graphene Oxide-Copper Nanocomposites Suppress Cariogenic *Streptococcus mutants* Biofilm Formation. Int. J. Nanomed..

[21] Chen X.W., Hai X., Wang J.H. (2016). Graphene/graphene oxide and their derivatives in the separation/isolation and preconcentration of protein species: A review. Anal. Chim. Acta.

[22] Li G.Y., Zeng J.X., Liu H.L., Ding P., Liang J.T., Nie X.M., Zhou Z.D. (2019). A fluorometric aptamer nanoprobe for alpha-fetoprotein by exploiting the FRET between 5-carboxyfluorescein and palladium ananoparticels. Microchim. Acta.

[23] Tan J., Lai Z.Q., Zhong L.P., Zhang Z.H., Zheng R., Su J., Huang Y., Huang P.P., Song H., Yang N. (2018). A Graphene Oxide-Based Fluorescent Aptasensor for the Turn-on Detection of CCRF-CEM. Nanoscale Res. Lett..

[24] Wen L.X., Lv J.J., Chen L., Li S.B., Mou X.J., Xu Y. (2019). A fluorescent probe composed of quantum dot labeled aptamer and graphene oxide for the determination of the lipopolysaccharide endotoxin. Microchim. Acta.

[25] Safavipour M., Kharaziha M., Amjadi E., Karimzadeh F., Allafchian A. (2020). TiO_2_ nanotubes/reduced GO nanoparticles for sensitive detection of breast cancer cells and photothermal performance. Talanta.

[26] Huang X., He Z., Guo D., Liu Y., Song J., Yung B.C., Lin L., Yu G., Zhu J.J., Xiong Y. (2018). Nanoquenchers to Enhance No-Wash Fluorescence Biosensors for Ratiometric Detection of Cancer Biomarkers. Theranostics.

[27] Li W.M., Bing T., Wei J.Y., Chen Z.Z., Shangguan D.H., Fang J. (2014). Cell-SELEX-based selection of aptamers that recognized distinct targets on metastatic colorectal cancer cells. Biomaterials.

[28] De Larrea C.F., Staehr M., Lopez A.V., Ng K.Y., Chen Y., Godfrey W.D., Purdon T.J., Ponomarev V., Wendel H.G., Brentjens R.J. (2020). Defining an Optimal Dual-Targeted CAR T-cell Therapy Approach Simultaneously Targeting BCMA and GPRC5D to Prevent BCMA Escape-Driven Relapse in Multiple Myeloma. Blood Cancer Discov..

[29] Shen Y., Li M., Liu T., Liu J., Xie Y., Zhang J., Xu S., Liu H. (2019). A dual-functional HER2 aptamer-conjugated, pH-activated mesoporous silica nanocarrier-based drug delivery system provides in vitro synergistic cytotoxicity in Her2-positive breast cancer cells. Int. J. Nanomed..

[30] Jamieson C., Martinelli G., Papayannidis C., Cortes J.E. (2020). Hedgehog Pathway Inhibitors: A new Therapeutic Class for the Treatment of Acute Myeloid Leukemia. Blood Cancer Discov..

[31] Zhao Y.J., Ma W.J., Zou S.Z., Chen B., Cheng H., He X.X., Wang K.M. (2019). Terminal deoxynucleotidyl transferase-initiated molecule beacons arrayed aptamer probe for sensitive detection of metastatic colorectal cancer cells. Talanta.

